# Financial Institutional and Market Deepening, and Environmental Quality Nexus: A Case Study in G-11 Economies Using CS-ARDL

**DOI:** 10.3390/ijerph191911984

**Published:** 2022-09-22

**Authors:** Usman Mehmood, Salman Tariq, Zia ul Haq, Ephraim Bonah Agyekum, Solomon Eghosa Uhunamure, Karabo Shale, Hasan Nawaz, Shafqat Ali, Ammar Hameed

**Affiliations:** 1 Remote Sensing, GIS and Climatic Research Lab, National Center of GIS and Space Applications, Centre for Remote Sensing, University of the Punjab, Lahore 54590, Pakistan; 2 Department of Political Science, University of Management and Technology, Lahore 54590, Pakistan; 3 Remote Sensing, GIS and Climatic Research Lab, National Center of GIS and Space Applications, Department of Space Science, University of the Punjab, Lahore 54590, Pakistan; 4 Department of Nuclear and Renewable Energy, Ural Federal University Named after the First President of Russia Boris Yeltsin, 19 Mira Street, Eka-Terinburg 620002, Russia; 5 Faculty of Applied Sciences, Cape Peninsula University of Technology, P.O. Box 652, Cape Town 8000, South Africa

**Keywords:** financial deepening, wavelet coherence, foreign direct investment, economic growth, CS-ARDL

## Abstract

This study presents a new insight into the dynamic relationship between financial institutional deepening (FID), financial deepening, financial market deepening (FMD), foreign direct investment (FDI), economic growth (GDP), population, and carbon dioxide emissions (CO_2_e) in the G-11 economies by employing a cross-sectionally augmented autoregressive distributed lag (CS-ARDL) approach during 1990–2019. The outcomes from the CS-ARDL and dynamic common correlated effects mean group (DCCEMG) models shows that financial deepening, GDP, FDI, and population degraded environmental quality both in the short run and the long run. Contrary to this, FID and FMD improves environmental quality in these countries. The government should work to maximize financial institutions (access, depth, efficiency) and financial markets (access, depth, efficiency) to reduce the CO_2_e. A strong positive and in-phase correlation of CO_2_e with economic growth and population is observed for G-11 countries. These results suggest policy makers should further improve financial institutions by creating opportunities for their populations. Moreover, the governments of G-11 countries should revise their foreign direct investment policies and attention should be given to import efficient means of energy production.

## 1. Introduction

The increasing impacts of climate change has emerged a consensus amid the social experts, energy and environmental scientists, and economists that the warming of the climatic system is obvious. This is obvious from the increasing global ocean and air average temperatures, ice and snow melting, and rising sea levels [1,2]. Apart from these, changing climate patterns also effect natural resources, ecosystems, and human wellbeing [3,4,5,6,7,8]. The increase in temperature is associated with increased concentrations of carbon dioxide (CO_2_), resulting in the deterioration of environmental quality [7,9,10,11]. Furthermore, the degradation of air quality also negatively impacts human health [12,13,14,15].

To meet the global energy demands of the growing population (POP), ~85% of the total energy is generated from fossil fuels, which contributes to ~57% of CO_2_ emissions (here and after, CO_2_e) [16,17,18]. The rate of CO_2_e from fossil fuels increased at 1.9% per year during 1970–2004, and it will continue to increase at 1.7% per year until 2030, while it will increase at 2% per year in the absence of effective policies [19]. Several researchers have recognized the distinct drivers of environmental loss, as well as how to maintain the world’s environment. For example, the Kyoto Agreement for the reduction of greenhouse gas emissions (GHGe) signed in 2005, and the 17th goal of SDGs clean energy initiative, were two big startups taken to resolve global climate disputes. Likewise, future goals have been set to lower GHGe by 45% and to keep the global temperature well below a 1.5 °C rise by 2030 and 2050, respectively.

Moreover, there are some other drivers that contribute to environmental deterioration, particularly in the developing world, e.g., economic growth, globalization, energy use, urbanization, transportation, industrial activities, and population [7,8,20,21]. These drivers have devastating environmental consequences. In this context, developing countries, including Pakistan, Ecuador, El Salvador, Georgia, Indonesia, Croatia, Jordan, Morocco, Honduras, Sri Lanka, and Paraguay (here and after, G-11), have made impressive economic developments over the past few years. However, this is done at the cost of various social and economic issues.

Multiple studies have highlighted the relationship amid various socioeconomic variables, air pollution scenarios, GHG emissions, and meteorological parameters [21,22,23,24,25,26,27,28,29,30,31,32,33,34,35,36,37,38,39]. For instance, Ref. [40] studied effects of globalization and tourism on CO_2_e in South Asian countries and found that globalization can improve air quality through innovations and advancements in technologies. They also found gross domestic products (GDP) and energy consumption (EC) enhances CO_2_e. Shahbaz et al. [41] found a positive relationship between GDP, foreign direct investment (FDI), and CO_2_e in North African and Middle Eastern economies. Essandoh et al. [42] found positive impacts of the FDI and CO_2_e in the long-run and long-run negative effects amid CO_2_e and trade in developing economies. Musah et al. [43] examined how the increase in the POP of the country led to more CO_2_e, while [16] found a decrease in CO_2_e with POP growth. Similarly, studies have also shown that financial development mitigate [44,45] and escalate [46,47] CO_2_e. Sadorsky et al. [48] examined how financial provisioning to the market results in enhanced economic happenings, thereby increasing CO_2_e.

The association amid financial growth and the quality of the environment is a minor criterion of financial sector performance. According to our understanding, a sole study has focused on the mobilizing influences of financial deepening on environmental dilapidation [11]. However, they ignore the impact of FID on CO_2_e. Therefore, this study aims to analyze the impact of financial deepening, financial institutional deepening (FID), financial market deepening (FMD), GDP, FDI, and POP on CO_2_e in G-11 economies during 1990–2019. This study contributes to existing literature by adding the relationships between financial deepening and CO_2_e for G-11 economies and individuals and offers comparative results and the effects of FID and FMD. We employ a cross-sectionally augmented autoregressive distributed lag (CS-ARDL) model to assess the long-run and short-run effects of financial deepening on CO_2_e. Furthermore, the wavelet coherence analysis is also included so to check the impact of independent parameters on the dependent parameter (CO_2_e). Firstly, this study examines the effects of financial deepening, financial market deepening, and financial institutional deepening on CO_2_e. Secondly, this study uses the CS-ARDL methodology to panel data for G-11 countries. Thirdly, the work also employs other parameters such as GDP, foreign direct investment, and population in G-11 economies from 1990 to 2019. Fourthly, this work uses the different wavelet coherence method so to explain the cause–effect relationship between the variables. This technique also describes the correlation (negative/positive) of the two different variables.

This study is divided as follows. The first segment incorporates the introduction, exploring the associations among financial deepening and CO_2_e. The second section provides a brief explanation of the dataset sources, methods, and models. The third section provides the results from the CS-ARDL and wavelet coherence techniques; lastly, the fourth section offers conclusions and some appropriate policy implications.

## 2. Theoretical Framework and Literature Review

The present work analyzes the linkages of financial deepening, financial institutional deepening, financial market deepening, GDP, FDI, and population on CO_2_e in G-11 economies during 1990–2019, since, to meet the energy demands for economic development, most of the energy is produced by fossil fuel burning, which results in elevated CO_2_e. Fossil fuels comprise coal, natural gas, and oil, which are the big contributors of CO_2_e since the Industrial Revolution. Therefore, considering the work of [11,23,25,31], we develop an econometric model to analyze the long-run effects of financial deepening, financial institutional deepening, financial market deepening, GDP, FDI, and population on CO_2_e.

Several studies analyze the association between FDI, FID, FMD, GDP, and population. For example, [49] investigated how, for East Asian countries, by increasing per capita GDP and FDI, CO_2_e increased from 2011 to 2020 in the short run while having an insignificant impact in the long run. Mehmood et al. [25] observed the positive influence of financial institutional deepening on CO_2_e by using the CS-ARDL method in the long run and short run in G-10 economies from 1990 to 2020. Akinci et al. [50] showed a positive effect of the GDP on CO_2_e in the long run by using the dynamic ordinary least squares (DOLS) technique in European Union countries from 2000 to 2017. Li et al. [11] observed that financial market deepening and financial institutional deepening have an adverse effect on environmental quality by increasing the amount of CO_2_e in BRICS countries from 1990 to 2019. Aminata et al. [51] found positive effects of GDP and population on CO_2_e by using the vector error correction model in China and India from 1984 to 2014 at shorter and longer scales, whereas [52] found the negative impacts of financial deepening on CO_2_e in GCC countries from 1993 to 2019. Mohsin et al. [53] found significant positive and negative relations between GDP and CO_2_e for Europe and Central Asia by using the ARDL method for the duration from 1971 to 2016. The authors presented a positive effect of the GDP and population on CO_2_e in 15 of the most densely populated developing countries for the duration 2002–2020 [54]. The authors of [55] stated a bidirectional causality between GDP growth and CO_2_e in the developing countries. Sane et al. [56] analyzed how GDP and CO_2_e are more relevant to each other because of how economic growth will increase CO_2_e due to industrial sector expansion. Aslam et al. [57] observed significant long-run relations between GDP and CO_2_e in Malaysia during 1971–2016 by using the ARDL method.

Latief et al. [58] reported an inverted U-shaped relationship of urban population and GDP with environmental degradation in Mediterranean economies during 2001–2016 by utilizing the generalized method of moments technique. Latief et al. [59] found a causation between urban population with CO_2_e and domestic capital with FDI in South Asian countries during 1990–2016. Sattar et al. [60] observed that the least-developed party economies meeting the goals of the Paris Agreement require financial assets and skills, as well as climate familiarity with proficiency.

Wang et al. [61] used the full modified ordinary least squares (FMOLS) and dynamic normal least squares (DOLS) models and found that FDI, the tourism sector, and environment-friendly electricity were positive contributors towards CO_2_e in China from 1980 to 2019. Mehmood et al. [23] examined the relationship of FDI with renewable energy and found a significant decrease in CO_2_e in South Asian countries by using CS-ARDL from 1996 to 2019. The authors of [62] analyzed the income-based division of 124 countries of the world by using the generalized method of moments analysis (GMM) and observed that FDI deteriorated the environmental quality by increasing CO_2_e in the lower middle-income countries from 1990 to 2018. Uzair et al. [63] found that CO_2_e had a decreasing trend for economic development while FDI showed an increasing trend for economic development in India, Pakistan, and Bangladesh during 1971–2014.

Awan et al. [64] reported that FDI positively affected environmental degradation at the 5th to 50th quantiles during 1996–2015 by using the moments quantile regression method. Weimen et al. [65] employed fully modified ordinary least squares (PFM-LS) and panel dynamic least squares (PD-LS) and found that FDI is in direct relation with CO_2_e, and that the situation is more distressing in the developing world. Sreenu et al. [66] applied the ARDL and nonlinear autoregressive distributed lag models and reported that FDI flow into India was highly dependent on less stringent environmental protection laws during 1990–2020. Yahong et al. [67] confirmed that GDP and population are associated with increased CO_2_e in Asian developing countries by employing the system-generalized method of movement during 2006–2017. Dkhili [68] observed a negative relationship between FDI and REC in Middle Eastern and North African countries during 1990–2018 by using the EKC hypothesis.

However, the effect of financial deepening, financial market deepening, financial institutional deepening, FDI, GDP, and population on CO_2_e was not analyzed. Therefore, this study investigates the association of the aforesaid variables by using CS-ARDL during the aforementioned study period in G-11 countries.

## 3. Datasets, Method, and Models

### 3.1. Econometric Methodology

The ever-increasing energy demands of developing countries are mostly dependent on nonrenewable energy sources such as fossil fuels that are the main cause of CO_2_e. FDI also increases the CO_2_e in developing countries due to less stringent environment protection laws. To analyze the effect of FDI, GDP, financial deepening, financial market deepening, financial institutional deepening, and population, we made use of an econometric model, CS-ARDL, for G-11 countries.

(1) $$CO_{2\left(i,t\right)}e=f\left(FDI_{\left(i,t\right)},GDP_{\left(i,t\right)},FD_{\left(i,t\right)},\;\;FMD_{\left(i,t\right)},\;FID_{\left(i,t\right)},\;\;POP_{\left(i,t\right)}\right)$$

where financial deepening and CO_2_ are the input and output series, correspondingly. Meanwhile FDI, GDP, FMD, FID, and POP are taken as limiting parameters in order to reduce the influence in the outputs. Financial development is well-defined as anodizing market size (depth), the capability of individuals to the admittance financial amenities (access) and the capability of institutions to give low-cost financial services with sustainable revenues, and the capital market activity level (efficiency). The pace of financial development countrywide matters a lot. At a higher speed, FID can lead to financial and economic turmoil, encouraging more risk-taking and high leverage [69]. Therefore, limiting at a good pace places a premium on developing excellent regulatory and institutional frameworks. The FID is created from financial institutional efficiency, access, and depth, while FMD is made from financial market depth, financial market access, and financial market efficiency [11]. Financial deepening refers to the increase or decrease in the supply of countries’ financial assets, e.g., developed economies have high financial deepening that leads to higher growth of economic and whole-country development [70]. On the other hand, financial development mobilizes savings, expands the allocation of resources, stimulates the exchange of information, and accelerates diversification and risk management. It also nurtures the financial stability and resilience of the country. Panel data from Equation (1) is as follows:

(2) $$\begin{array}{ll} CO_{2\left(i,t\right)}e=\Phi_{i,t} & +\;\gamma_{1}FDI_{i,t}+\;\gamma_{2}GDP_{i,t}+\;\gamma_{3}FD_{i,t}+\;\gamma_{4}FMD_{i,t}+\gamma_{5}FID_{\left(i,t\right)}\\ & +\gamma_{6}POP_{\left(i,t\right)}+\;\mu_{i,t} \end{array}$$

where $\gamma_{1},\;\;\gamma_{2},\;\;\gamma_{3},\;\;\gamma_{4},\;\;\gamma_{5}$, and $\gamma_{6}$ represents the parameters of foreign direct investment, GDP, financial deepening, FMD, FID, and population, respectively, of a country (*i*) in time *t*, while $\mu_{i,t}\;\mathrm{and}\;\;\Phi_{i,t}\;\;\;$ represent the residual and constant terms, respectively. Panel data is transformed into the log form to avoid multicollinearity [71,72]. Thus, Equation (2) becomes:

(3) $$\begin{array}{ll} CO_{2\left(i,t\right)}e=\Phi_{i,t} & +\gamma_{1}lnFDI_{i,t}+\gamma_{2}lnGDP_{i,t}+\gamma_{3}lnFD_{i,t}+\gamma_{4}lnFMD_{i,t}\\ & +\gamma_{5}lnFID_{\left(i,t\right)}+\gamma_{6}lnPOP_{\left(i,t\right)}+\mu_{i,t} \end{array}$$

### 3.2. Model Building

The cross-sectional dependence (CSD) test of [73] is used to verify the dependencies in residual terms. The cross-sectionally augmented Dickey–Fuller (CADF) and CIPS unit root tests are applied to verify the stationarity parameters properties, whereby the test is employed to check the homogeneity in the slope parameters. The test designed by [74] is used to assess the cointegration characteristics of the series. Subsequently, the CS-ARDL model designed by [75] and the DCCEMG model proposed by [75] are used to control the endogeneity and heterogeneity in the slope. This study opted for a methodology that has the ability to disclose potential endogeneity problems, i.e., the CS-ARDL model. The CS-ARRDL is vigorous in the presence of misspecification bias, CSD, nonstationarity, serial correlation of error terms, and the endogeneity bias issues [76]. The robustness of the CS_ARDL is validated by using the DCCEMG approach. The DCCEMG method can deal with the problems of multicollinearity.

The CSD residuals were utilized to measure the flexible impact of the independent variables on the dependent parameters. In addition to the CS-ARDL model, cross-sectional averages (CSA) are used to limit cross-sectional correlation, specified as:

(4) $$\begin{array}{ll} lnCO_{2\left(i,t\right)}e=\;\Phi_{i} & +\overset{p_{y}}{\underset{j\;=\;1}{\sum }}\lambda_{ij}lnCO_{2\left(it-j\right)}e+\;\overset{p_{x}}{\underset{j\;=\;0}{\sum }}\gamma_{1j}lnFDI_{\left(it-j\right)}+\;\overset{p_{x}}{\underset{j\;=\;0}{\sum }}\gamma_{2j}lnGDP_{\left(it-j\right)}\\ & +\overset{p_{x}}{\underset{j\;=\;0}{\sum }}\gamma_{3j}lnFD_{\left(it-j\right)}+\;\overset{p_{x}}{\underset{j\;=\;0}{\sum }}\gamma_{4j}lnFMD_{\left(it-j\right)}\\ & +\;\overset{p_{x}}{\underset{j\;=\;0}{\sum }}\gamma_{5j}lnFID_{\left(it-j\right)}+\overset{p_{x}}{\underset{j\;=\;0}{\sum }}\gamma_{6j}lnPOP_{\left(it-j\right)}\\ & +\;\overset{p}{\underset{j\;=\;0}{\sum }}\psi_{1j}{\bar{lnCO}}_{2\left(t-j\right)}e+\;\;\;\overset{p\;}{\underset{j\;=\;0}{\sum }}\psi_{2j}{\bar{lnFDI}}_{\left(t-j\right)}\\ & +\overset{p}{\underset{j\;=\;0}{\sum }}\psi_{3j}{\bar{lnGDP}}_{\left(t-j\right)}+\;\overset{p}{\underset{j\;=\;0}{\sum }}\psi_{4j}{\bar{lnFD}}_{\left(t-j\right)}\\ & +\;\;\overset{p\;\;}{\underset{j\;=\;0}{\sum }}\psi_{5j}{\bar{lnFMD}}_{\left(t-j\right)}+\overset{p}{\underset{j\;=\;0}{\sum }}\psi_{6j}{\bar{lnFID}}_{\left(t-j\right)}\\ & +\;\;\overset{p}{\underset{j\;=\;0}{\sum }}\psi_{7j}{\bar{lnPOP}}_{\left(t-j\right)}+\;\;\mu_{it} \end{array}$$

where ${\bar{lnCO}}_{2\;}e$,$\bar{lnFDI}$, $\bar{lnGDP}$, $\bar{lnFD}$, $\bar{lnFMD}$, $\bar{lnFID},\;\;\bar{lnPOP\;}$ depicts the CSA of the dependent and the independent variables $;\;\Phi_{i}$ and $\lambda_{ij}\;$ represents the effect of the specifications of the unexplored countries and the lagged coefficients of the response parameters, respectively; $\gamma_{1j}$,…,$\gamma_{6j\;}$ and $\psi_{1j},\ldots .,\psi_{7j}$ are the parameters of the covariates and the CSA of the delayed series. The hardiness of the CS-ARDL model is evaluated by using the DCCEMG technique. The DCCEMG approach is consistent with the technique of [75], expressed as:

(5) $$\begin{array}{ll} lnCO_{2\left(i,t\right)}e=\;\Phi_{i} & +\;\lambda_{i}lnCO_{2\left(i,t-1\right)}e+\;\gamma_{1}lnFDI_{i,t}+\gamma_{2}lnGDP_{i,t}\;+\gamma_{3}lnFDx_{i,t}\;\\ & +\;\;\gamma_{4}lnFMD_{i,t}+\gamma_{5}lnFID_{i,t}\;\gamma_{6}lnPOP_{i,t}\\ & +\overset{K}{\underset{r\;=\;0}{\sum }}\Phi_{1ir}{\bar{lnCO}}_{2\left(i,\;\;t-1\right)}e+\overset{K}{\underset{r\;=\;0}{\sum }}\Phi_{2ir}{\bar{lnFDI}}_{\left(i,\;\;t-1\right)}\\ & +\;\overset{K}{\underset{r\;=\;0}{\sum }}\Phi_{3ir}{\bar{lnGDP}}_{\left(i,\;\;t-1\right)}+\;\overset{K}{\underset{r\;=\;0}{\sum }}\Phi_{4ir}{\bar{lnFDx}}_{\left(i,\;\;t-1\right)}\\ & +\;\overset{K}{\underset{r\;=\;0}{\sum }}\Phi_{5ir}{\bar{lnFMD}}_{\left(i,\;\;t-1\right)}+\;\;\overset{K}{\underset{r\;=\;0}{\sum }}\Phi_{6ir}{\bar{lnFID}}_{\left(i,\;\;t-1\right)}\\ & +\;\overset{K}{\underset{r\;=\;0}{\sum }}\Phi_{7ir}{\bar{lnPOP}}_{\left(i,\;\;t-1\right)}+\;e_{it} \end{array}$$

### 3.3. Datasets Sources and Descriptive Statistics

The panel datasets of foreign direct investment, GDP, financial deepening, FMD, FID, and population for the G-11 economies during 1990–2019 is used in this study. The study period, based on data availability for G-11 countries, is restricted from 1990 to 2019. The detail of the datasets, along with their sources, is given in Table 1.

We selected G-11 countries for our research work because, firstly, these are mostly lower middle-income countries under the burden of debt, which can reduce their sustainable growth. Secondly, GDP growth is more dependent on fossil fuels, which are degrading the environmental quality. Thirdly, they are more vulnerable to climate change effects.

The summary statistics comprising maximum, minimum, mean, standard deviation (SD), skewness, and kurtosis of all the aforesaid variables is given in Table 2. The maximum mean value is for GDP as (1.17 × 10^11^ ± 2.22 × 10^11^), followed POP (4.38 × 10^7^ ± 8.23 × 10^7^), CO_2_e (69,817.14 ± 136,385.24), FDI (3.64 ± 3.79), FID (0.31 ± 0.09), financial deepening (0.23 ± 0.13), and FMD (0.16 ± 0.19). The distribution of the entire series is positively skewed. For kurtosis, the distributions of CO_2_e, FDI, GDP, and POP are leptokurtic in shape, while those of financial deepening, FID, and FMD are platykurtic in shape.

**Figure 1:**
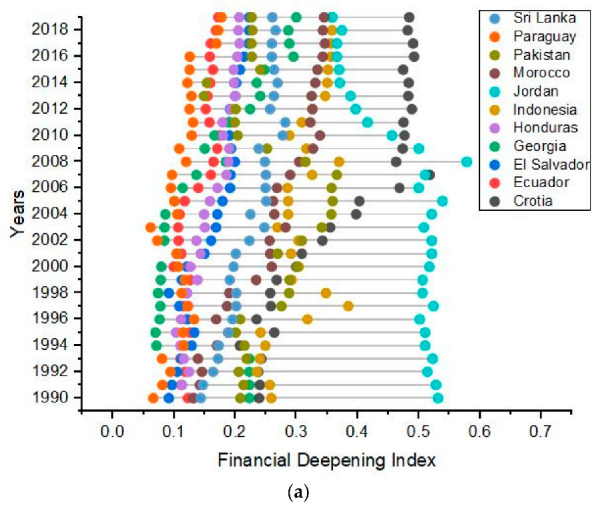

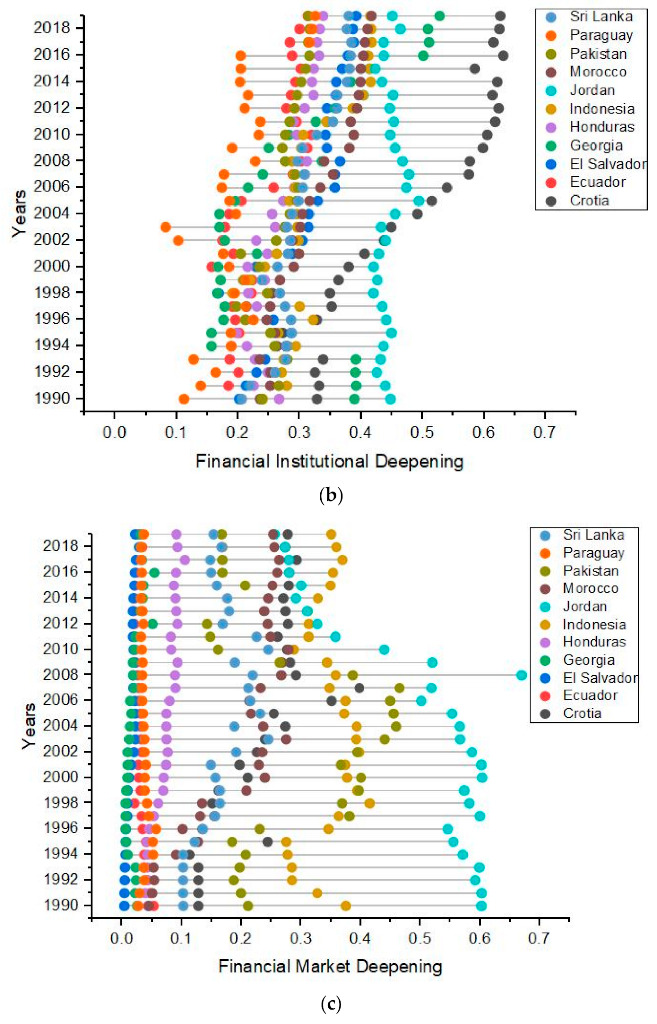

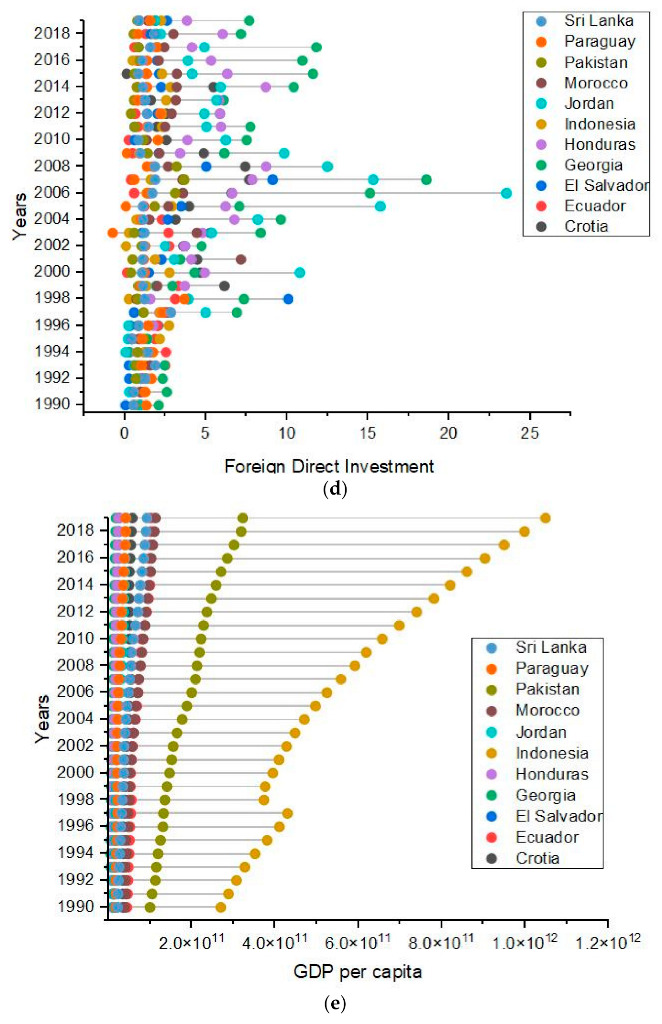

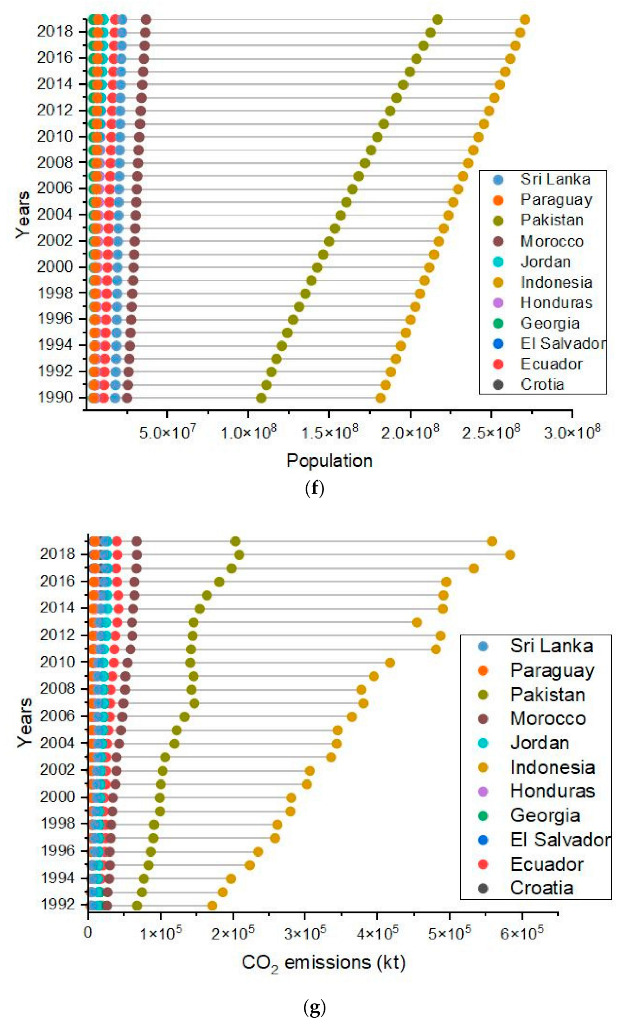
Temporal variations in (**a**) financial deepening, (**b**) FID, (**c**) FMD, (**d**) FDI, (**e**) GDP, (**f**) population, and (**g**) CO_2_e for G-11 countries during 1990–2019.

Furthermore, the annual variations in financial institutional deepening (FID), financial market deepening (FMD), financial deepening (FD), GDP, FDI, and population is given in Figure 1. Figure 1a shows an increasing and decreasing trend of financial deepening for Croatia and Jordan, respectively, though the financial institutional deepening is increasing and fluctuating for Croatia and Jordan, respectively, as shown in Figure 1b. Figure 1a shows that Jordan has the highest financial deepening, reaching its peak value in 2008 and then decreasing until 2019. Jordan had the highest value of financial market deepening and FDI in 2008 and 2006, respectively, as shown in Figure 1c,d, respectively. The GDP, population, and CO_2_e of all the countries are increasing throughout the duration, and Pakistan and Indonesia have exceptional values, as shown in Figure 1e–g. The highest values of CO_2_e for Pakistan and Indonesia were in 2018, as shown in Figure 1g. The highest value of FDI was observed for Georgia and Jordan during 2004–2008. Pakistan and Indonesia have the highest economic growth and population, which resulted in an elevated CO_2_e in these countries during the study period (Figure 1e–g). The highest value of the FDI was observed for Georgia and Jordan during 2004–2008. Pakistan and Indonesia have highest economic growth and population, which resulted in an elevated CO_2_e in these countries during the study period (Figure 1e–g).

## 4. Results and Discussion

### 4.1. Cross-Sectional Dependence and Heterogeneity Test Results

Prior to checking the stationarity properties of the data [77], a test is applied to check the CSD amid the series, the results of which are displayed in Table 3. From the table, the outcomes discard the null hypothesis of no CSD amongst the residuals of the model, indicating that an impact on one economy could deluge the rest of the countries. This finding collaborates with that of [78] for G-11 economies, with [79] for West Africa, with [16] for South Asian economies, with [23] for Sri Lanka, Pakistan, India, Bangladesh, Nepal, and Bhutan, but contradicts [80] for Northern China.

Furthermore, neglecting heterogeneity in the slope coefficients could lead to bias [81]. Hence, following the studies of [8,79,81], this study employs the test devised by [82] to verify the heterogeneity in the slope parameters. The results obtained from the Pesaran and Yamagata, 2008 test, as shown in Table 4, reveal that there exists diversity in the slope variables, suggesting considerable disparity in the G-11 countries. The test by [82] is superior to the other conventional homogeneity techniques in that it incorporates the CSD both in the long run and the short run. Moreover, this test is appropriate for a longer period and smaller sample size. The empirical outcomes of [83] for the G-7 nations, and for [81] in North Africa and [79] in Africa, support the overhead revelations.

### 4.2. Unit Root Test and Co-Integration Test Results

After checking the CSD and slope heterogeneity amid the series, the Westerlund [84] test is employed to perform the cointegration analysis. The Gt and Ga denote the mean information of a group, while Pt and Pa signifies the overall panel information. The outcomes of the Westerlund [84] test, as shown in Table 5, revealed the long-run relationship among the parameters. The research conducted by [83,85] for G-7 countries, by [86] for the United Kingdom, by [43] for NAFTA economies, by [40] for South Asian countries, and by [87] for G-20 countries lends support to the above outcomes.

Afterwards, we used the cross-sectionally augmented Dickey–Fuller (CADF), Shin (CIPS) test, Pesaran, and cross-sectional Im to assess the stationarity belongings of the parameters. The results presented in Table 6 show that the null hypothesis of no stationarity for the whole series could not be denied at the levels but for the first difference. These inferences also indicate the homogeneous order of integration at the first difference amid the parameters. This is the reason behind why the CS-ARDL approach is employed to evaluate the long-run relationship within the whole series. Likewise, this finding was presented by [83] for G-7 economies, by [11] for BRICS economies, by [23] for three of the developing economies of Asia, and by [88] for South Asia.

### 4.3. CS-ARDL

The CS-ARDL model is applied to assess the robust effects of the FID, FMD, GDP, FDI, financial deepening, and population on CO_2_e in G-11 nations. The outcomes displayed in Table 7 reveal that a 1% increase in financial deepening will worsen the air quality by 0.5269% in the long run and 0.8959% in the short run amongst G-11 countries. Sheraz et al. [89] observed the positive effect of financial development on CO_2_e in the BRI countries by using the generalized method of moments (GMM) method. The financial market deepening and financial institutional deepening, on the other hand, improves the environmental quality. For instance, a 1% increase in financial market deepening and financial institutional deepening will decrease the environmental quality by 0.0549% and 0.7626% in the short run, and 0.0393% and 0.4458% in the long run. Habiba and Xinbang [90] found that financial market development and financial institutional development, and their indices, improved the environmental quality in European and Sub-Saharan African countries. Li et al. [11] reported that FID and FMD decreases CO_2_e by 0.766% and 2.323%, respectively, under positive shocks by using the NARDL-PMG models, while financial deepening increases CO_2_e by 3.073% under negative shocks in BRICS countries. The FID and FMD decreases the CO_2_e by giving easy access to green credits in Turkey [91]. This implies that financial institutional and market deepening encourages a convenient approach to green credits for enhancing the green economy, thereby reducing the degradation of the environment. In short, financial deepening can improve the availability and accessibility of green technology for organizations and individuals, which can continue to help these countries to achieve net zero carbon emissions on a global scale.

However, the impact of GDP, FDI, and population is positive on CO_2_e for G-11 economies. A 1% increase in GDP, FDI, and population will lead to 0.6264%, 0.0090%, and 0.9012% increase in CO_2_e in the long run and 1.1230%, 0.0140%, and 1.4735% in the short run, respectively. Mohsin et al. [53] also found that CO_2_e is the greater cause of GDP increase, and the FDI increased CO_2_e in Europe and Central Asia. The authors of [55] observed that a 1% increase in economic growth increased the CO_2_e by 0.17% in the long run by availing the autoregressive distributed lag method for developing countries. Abid et al. [92] reported a unidirectional causality between FDI and CO_2_e in G-8 countries. Rehman et al. [93] found a positive association between economic growth, population growth, and CO_2_e in Pakistan. GDP and financial deepening were in direct relation to CO_2_e, i.e., an increase in GDP led to an increase in the money assets of people, which allowed people to buy more environmentally friendly products and hence lessen CO_2_e [94]. Faisal et al. [91] observed that financial development provides finance, which boosted eco-friendly technology usage. Financial deepening can decrease the CO_2_e into the atmosphere by encouraging renewable resources [95]. Le et al. [96] found that higher FDI led to higher CO_2_e. They also argue that strong financial markets could act as catalysts for the application of energy-efficient clean technologies. Musah et al. [97] also found that an increase in economic growth led to higher risks of environmental pollution for North African countries. Furthermore, our results also support the findings of [7].

The outcomes attained from the DCCEMG technique, portrayed in Table 8, illustrate the toughness of the CS-ARDL method. The assessed values of the parameters may vary in significance and weightiness, but the like signs of the two techniques shows robustness of the process. Likewise, comparable movements in the post estimated data further put stress on the effectiveness and trustworthiness of this study.

### 4.4. Wavelet Coherence

**Figure 2:**
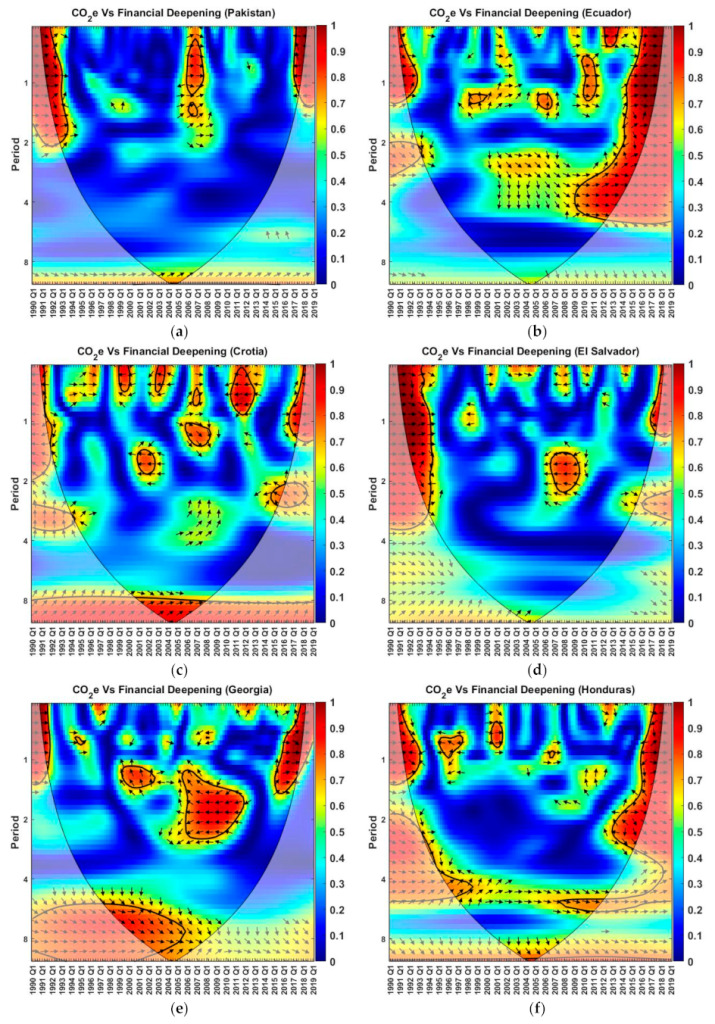

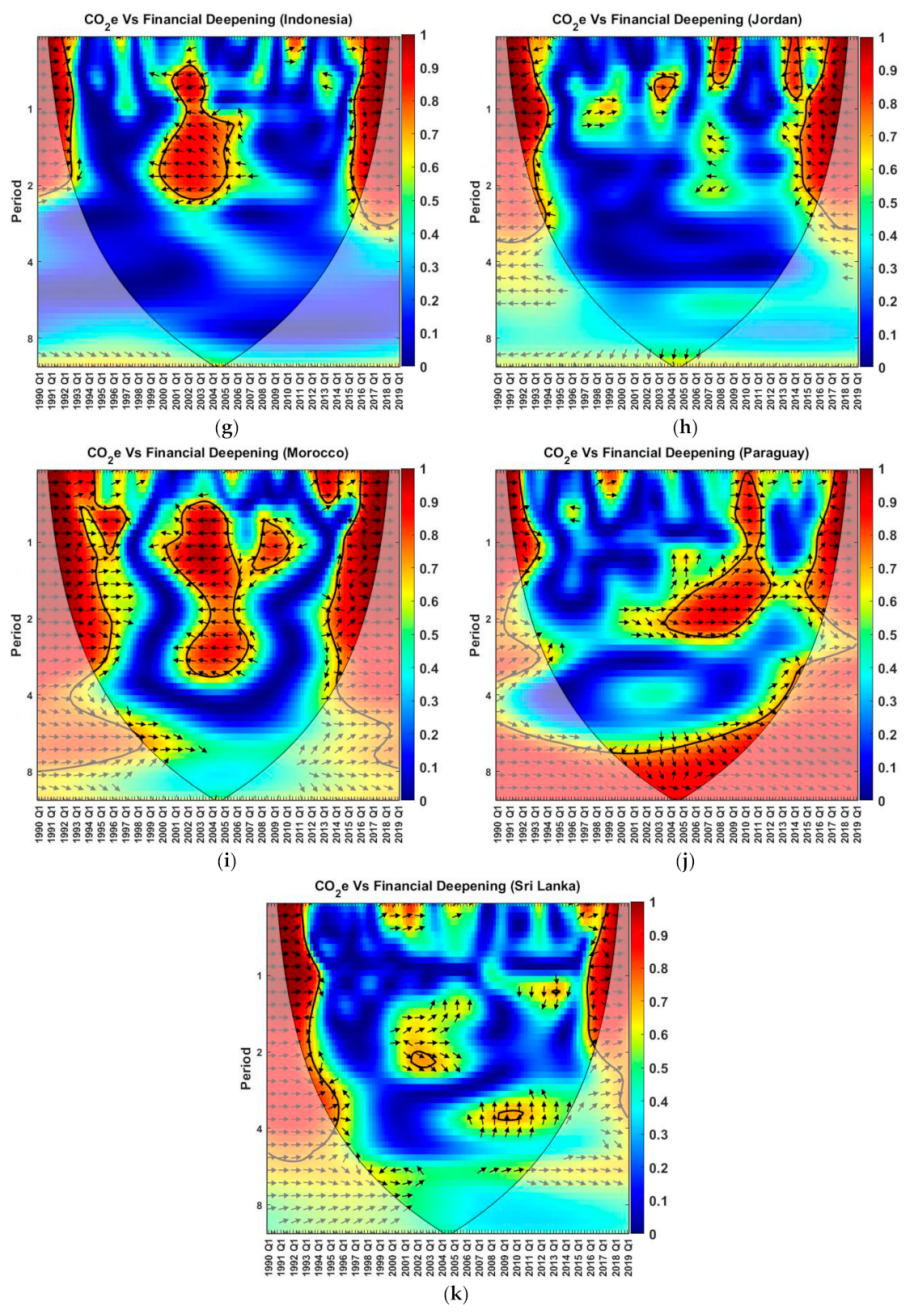
The wavelet coherence between CO_2_e and financial deepening for (**a**) Pakistan, (**b**) Ecuador, (**c**) Croatia, (**d**) El Salvador, (**e**) Georgia, (**f**) Honduras, (**g**) Indonesia, (**h**) Jordan, (**i**) Morocco, (**j**) Paraguay and (**k**) Sri Lanka from 1990 to 2019.

The wavelet coherence analysis of CO_2_e with financial deepening for G-11 countries from the first quarter of 1990 to the first quarter of 2019 is displayed in Figure 2. The outputs of the wavelet coherence helps to show how much the magnitude of one parameter will effect another [98]. The Morlet wavelet approach is used in the wavelet–power spectrum. The frequency is divided into four bands: 0 to 1, 1 to 2, 2 to 4, and 4 to 8. These frequency bands can be depicted as very-short-run, short-run, medium-run, and long-run cycles [99,100].

The wavelet coherence method is used to acquire the correlation between the two-time series in a time and time period domain. Time is on the *x*-axis and the time period is on the *y*-axis. The coherence level is from 0 (minimum) to 1.0 (maximum) in dark blue to dark red colors, respectively. The black line contours highlight the areas of high correlation with a 5% significance level by utilizing Monte Carlo simulations. The area from the cone of influence to the axes is affected by the edge effects and does not consider it as reliable [101]. The arrows in the contours show the mutual directions of the two variables. The arrows to the right and left directions manifest the two parameters and are in phase and antiphase with respect to each other, respectively, while between both orientations show the lead–lag relationship, such as right–upward and left–downward; the second variable leads the first one with left–upward and right–downward; the first variable leads the second one [102].

All the countries showed a high correlation around the starting and ending years at higher frequencies. Furthermore, the starting years are mostly in phase, and ending years are in mixed order, i.e., both in-phase and antiphase. The wavelet coherence plot for Pakistan shows a high correlation between the variables from a 0 to 2 scale during the years from 1991 to 1992, 2006 to 2007, and 2017 to 2018, as shown in Figure 2a. From 1991 to 1992 and 2006 to 2007, the arrows are mostly in phase, but from 2017 to 2018, they are in antiphase. This shows that CO_2_e and FD were moving in the same direction from 1991 to 1992 and 2006 to 2007 but were moving in the opposite direction in comparison to each other from 2017 to 2018. The authors of [103] revealed significant dependence of CO_2_e on economic growth and urbanization by using the wavelet coherence approach for Malaysia.

Figure 2b shows the coherence plot for Ecuador from 1991 to 1992 and 2009 to 2018, with a high in-phase correlation for periods from 0 to 1 and 0 to 6, respectively. From 1998 to 1999 and 2005 to 2006, the left–upward (CO_2_e leading FD) and right–upward (FD leading CO_2_e) relations exist, respectively, on a scale from 1 to 2. The authors showed a positive association between CO_2_e and financial development in Thailand by utilizing wavelet plot. They observed the one-way relationship from the CO_2_e to economic progress via financial development in the Ecuadorian economy. Figure 2c shows, in Croatia from 1999 to 2000, 2003 to 2004, 2011 to 2013, and 2017 to 2018, an antiphase correlation between the variables for the 0 to 1 scale, and from 2001–2002 in the 1 to 2 scale. A highly in-phase correlation was also present around period 8 from 2001 to 2007, while from 2006 to 2007, an FD-leading CO_2_e relation was also present from the 0 to 1 scale. The authors of [104] reported that economic growth led to an increased CO_2_e over Thailand between 1971 and 2018 by using the wavelet coherence approach.

A significant in-phase and antiphase connection can be seen in El Salvador, Figure 2d, for the 0 to 4 period from 1991 to 1993 and 2007 to 2008, respectively. For Georgia, from 1991 to 1992 and 2016 to 2018, an in-phase relation can be seen for the 0 to 1 scale, and on the other side, antiphase arrows can be seen from 2006 to 2011 in between the 1 and 4 scale. From 1999 to 2002 and 1998 to 2005, it also exhibited a CO_2_e-leading phenomenon from the 1 to 2 and 6 to 10 scales, respectively. Honduras showed FD leading from 1996 to 1998 and from 2003 to 2011, and CO_2_e leading for the periods from 4 to 12. Furthermore, other areas did not show a determinant correlation. Indonesia displayed high correlation values (0.9–1.0) from 2000 to 2005, with an antiphase relation from the 0 to 2 period of time, while Jordan had a red blob from 2007 to 2009 at a higher frequency besides the edge years’ high values.

An in-phase and antiphase relation from the 0 to 4 scale from 1991 to 1995 and from 2001 to 2006, respectively, for Morocco is shown in Figure 2i. The authors of [105] examined showed that the financial development impacted CO_2_e positively in South Africa by using the wavelet coherence technique. Paraguay also had a positive in-phase relation from 1991 to 1992, 2004 to 2011, and 2017 to 2018 in between the 0 and 2 scale. At the lower frequencies, CO_2_e led the FD for Paraguay. A positive relationship was also shown between the variables at the starting years for Sri Lanka for the 0–4 scale, while a negative one was shown for the ending years. Furthermore, CO_2_e led the FD around the period value 2 from 2001 to 2003. Comparable results were found by [106,107] for China, and by [38] for G-7 economies.

## 5. Conclusions

This study examines the associations among CO_2_e, FDI, financial deepening, financial market deepening, financial institutional deepening, GDP, and population in G-11 countries by employing the CS-ARDL model during 1990–2019. The efficacy and vigorousness of the CS-ARDL approach is validated by using the DCCEMG method. We also employ the CSD, CADF, and CIPS unit root and slope heterogeneity tests. Furthermore, the Westerlund (2007) test is used to examine the diversity in slope that approves the cointegration association among the variables. Lastly, the wavelet coherence approach is applied to carry out localized analysis by capturing local sub-image regions of a broader picture.

The outcomes of the CS-ARDL reveals that enhancing financial deepening will deteriorate environmental quality in G-11 countries. For example, a 1% increase in financial deepening will worsen the air quality by 0.5269% in the long run and 0.8959% in the short run. Since financial development enhances the economic growth of a country, which is associated with increased energy demands, the governments of G-11 economies must therefore focus on the allocation of resources and diversification of the facilities for the achievement of environmental sustainability.

Contrary to this, an increase in financial market deepening and financial institutional deepening enhanced the air quality, i.e., for a 1% increase in financial institutional deepening and financial market deepening, there will be decrease of 0.4458% and 0.0393% in the long run and 0.7626% and 0.0549% in the short run, respectively. Therefore, policymakers should focus on increasing public awareness, imposing strict laws to limit CO_2_e, and providing access to financial services at a low cost and with sustainable revenues for improving environmental quality.

Furthermore, the GDP, FDI, and population had positive impact on CO_2_e for G-11 economies, both in the short run and the long run. Since a high population number means high energy demands, which in turn increases CO_2_e due to the burning of fossil fuels, oil, gas, coal, and other fuels, governments of G-11 countries must encourage cleaner and greener energy production to achieve environmental sustainability.

The wavelet coherence analysis between CO_2_e and FD for Indonesia and Morocco showed an antiphase correlation from 2000 to 2006, while Paraguay showed an in-phase correlation from 2004 to 2011. The FD and CO_2_e are inversely related to each other for Georgia, Jordan, and El Salvador, and directly related for Pakistan and Honduras from 2006 to 2007.

### Policy Recommendations

The results reveal that financial market deepening (financial market access, financial market efficiency, and financial market depth) and financial institutional deepening (financial institutional access, financial market efficiency, and financial market depth) are environmentally friendly in the estimated countries. These findings are important for policy makers so to further improve financial institutions such as law enforcement, education, and waste management. These institutions should encourage low-carbon-intensive industries and increase the public awareness so to further enhance the environmental quality.

As financial deepening in the G-11 countries increases the CO_2_e, the role of financial deepening must be improved so to attain a sustainable development. In this regard, the banks should offer loans to install renewable-energy sources. Likewise, companies and firms which produce environment friendly energy should be encouraged by giving them tax-free incentives and interest-free loans. While giving loans for cars, houses, and other electronic appliances, the motive should be encouraging users to buy fuel-efficient products.

Moreover, the FDI does not enhance the air quality, which means that these countries need to revise the foreign direct investment policies at the cost of environment, and attention should be given to import efficient means of energy production.

This study has a few limitations. The present work is restricted to G-11 economies, and similar studies should be continued to other groups of countries, probably by employing the panel NARDL method. This study mainly focused on the long-run and short-run effects of financial deepening, financial market deepening, and financial institutional deepening on environmental quality. Our study did not control other related environmental quality parameters such as environmental policy stringency, REC, and globalization.

Future research can expand on this work by adding other variables such as environmental policy stringency, REC, globalization, imports, exports, and technology innovation in the green growth model. Further research can be conducted at the microlevel in highly polluted economies. To obtain distinctive and consistent empirical results for the designing of efficient environmental and financial policies, research should focus on more advanced econometric techniques.

## Figures and Tables

**Table 1:** The parameters under investigation and their sources.

Parameters	Abbreviation	Measurement	Source
Carbon Dioxide emissions	CO_2_e	Kilo tons	World Bank
Population	POP	Total population	World Bank
Gross Domestic Products	GDP	Per capita (constant 2015 USD)	World Bank
Foreign Direct Investment	FID	Percentage of GDP	World Bank
Financial Deepening	FD	Index representing financial market growth	IMF
Financial Institutional Deepening	FID	Financial Institutional Access, depth and efficiency	IMF
Financial Market Deepening	FMD	Financial Market Access, depth and efficiency	IMF

**Table 2:** Represents the descriptive figures of the parameters.

Parameters	Mean	Minimum	Maximum	SD	Skewness	Kurtosis
CO_2_e	69,817.14	2380	583,110	136,385.24	2.31	4.15
FDI	3.64	0.04	23.53	3.79	2.07	5.60
FD	0.23	0.07	0.58	0.13	0.98	−0.05
FMD	0.16	0.003	0.67	0.19	1.09	−0.22
FID	0.31	0.15	0.53	0.09	0.23	−0.96
GDP	1.17 × 10^11^	4.69 × 10^9^	1.05 × 10^12^	2.22 × 10^11^	2.50	5.60
POP	4.38 × 10^7^	3.57 × 10^6^	2.71 × 10^8^	8.23 × 10^7^	1.86	1.64

**Table 3:** Results achieved for CSD analysis.

Parameter	Test Statistics (*p*-Value)
CO_2_e	40.583 *** (0.00)
FDI	10.413 *** (0.00)
FD	39.630 *** (0.00)
FMD	38.698 *** (0.00)
FID	39.883 *** (0.00)
GDP	40.619 *** (0.00)
POP	40.618 *** (0.00)

*** = is significant at 1%.

**Table 4:** Slope heterogeneity test.

Statistics	Test Value (*p*-Value)
Delta-tilde	3.427 *** (0.00)
Delta-tilde Adjusted	4.160 *** (0.00)

*** = significant at 1%.

**Table 5:** (Westerlund, 2007) test.

Dependent Variable: CO_2_e		
	Value	*p*-Value
Gt	−2.347	0.954
Ga	−5.101 **	0.040
Pt	−5.884	0.993
Pa	−7.111 *	0.089

** = significant at 5% and * = significant at 10%.

**Table 6:** CADF and CIPS Test.

Variables	CIPS Test		CADF Test	
	At Level	First Difference	At Level	First Difference
CO_2_e	−2.09	−5.39 ***	−2.03	−3.57 ***
FDI	−3.16	−6.03 ***	−2.52	−4.85 ***
FD	−2.26	−5.50 ***	−2.07	−4.08 ***
GDP	−2.40	−4.09 ***	−2.87	−3.73 ***
FMD	−2.56	−5.28 ***	−2.44	−4.17 ***
FID	−2.63	−5.29 ***	−2.46	−3.81 ***
POP	−1.80	−3.29 ***	−2.88	−3.76 ***

*** is significant at 1%

**Table 7:** CS-ARDL.

Dependent Parameter: CO_2_e						
Parameter	Short Run			Long Run		
	Coefficient	Standard Error	Significance	Coefficient	Standard Error	Significance
ΔlnCO_2_e	−0.7951 ***	0.0779	0.00	−1.7951 ***	0.0779	0.00
ΔlnFDI	0.0140 *	0.0103	0.07	0.0090 *	0.0069	0.09
ΔlnFD	0.8959 ***	0.7010	0.00	0.5269 **	0.3529	0.03
ΔlnFMD	−0.0549 *	0.1327	0.07	−0.0393 *	0.0701	0.07
ΔlnFID	−0.7626 *	0.5451	0.06	−0.4458 ***	0.2747	0.00
ΔlnGDP	1.1230 ***	0.1919	0.00	0.6264 ***	0.1045	0.00
ΔlnPOP	1.4735 ***	0.9041	0.00	0.9012 **	0.5649	0.01

*** significant at 1%, ** significant at 5%, * significant at 10%.

**Table 8:** Outcomes of the DCCEMG approach.

Variable	DCCEMG		
	Coefficients	Standard Error	Significance
FDI	0.0102 **	0.0042	0.015
FD	0.4199 *	0.3985	0.092
FMD	−0.0099 **	0.0923	0.014
FID	−0.3421 **	0.2827	0.026
GDP	0.9054 ***	0.2247	0.000
POP	2.5265 *	1.4022	0.072

*** significant at 1%, ** significant at 5%, * significant at 10%.

## Data Availability

Sources of data used for the study are provided in the text.
